# Optimum birth interval (36–48 months) may reduce the risk of undernutrition in children: A meta-analysis

**DOI:** 10.3389/fnut.2022.939747

**Published:** 2023-01-13

**Authors:** James Ntambara, Wendi Zhang, Anni Qiu, Zhounan Cheng, Minjie Chu

**Affiliations:** Department of Epidemiology, School of Public Health, Nantong University, Nantong, Jiangsu, China

**Keywords:** birth interval, undernutrition, underweight, stunting, wasting

## Abstract

**Background:**

Although some studies have highlighted short birth interval as a risk factor for adverse child nutrition outcomes, the question of whether and to what extent long birth interval affects better nutritional outcomes in children remains unclear.

**Methods:**

In this quantitative meta-analysis, we evaluate the relationship between different birth interval groups and child nutrition outcomes, including underweight, wasting, and stunting.

**Results:**

Forty-six studies with a total of 898,860 children were included in the study. Compared with a short birth interval of <24 months, birth interval of ≥24 months and risk of being underweight showed a U-shape that the optimum birth interval group of 36–48 months yielded the most protective effect (OR = 0.54, 95% CI = 0.32–0.89). Moreover, a birth interval of ≥24 months was significantly associated with decreased risk of stunting (OR = 0.61, 95% CI = 0.55–0.67) and wasting (OR = 0.63, 95%CI = 0.50–0.79) when compared with the birth interval of <24 months.

**Conclusion:**

The findings of this study show that longer birth intervals (≥24 months) are significantly associated with decreased risk of childhood undernutrition and that an optimum birth interval of 36–48 months might be appropriate to reduce the prevalence of poor nutritional outcomes in children, especially underweight. This information would be useful to government policymakers and development partners in maternal and child health programs, especially those involved in family planning and childhood nutritional programs.

## Introduction

Despite significant progress in reducing child mortality attributed to undernutrition, childhood undernutrition remains a major public health concern in developing countries. Undernutrition is most often measured by anthropometry and evaluated in terms of underweight, stunting, and wasting (1). These undernutrition indices are classified according to the World Health Organization (WHO) classification using child growth standard medians in terms of standard deviations (SDs) (2). Weight-for-age, height-for-age, and weight-for-height provide different information about the cognitive growth and body composition of children. Stunting (low height-for-age) captures early chronic exposure to undernutrition, wasting (low weight-for-height) captures acute undernutrition, and underweight (low weight-for-age) is a composite indicator that includes elements of stunting and wasting (3). Undernutrition, especially stunting, in the first 1,000 days of life, is associated with fewer neural connections in the brain, leading to poor cognitive development, and this damage is irreversible (4). Therefore, more attention should be paid to undernourished children to avoid the adverse health effects of this irreversible damage on their future growth and development.

The WHO 2025 global nutrition target is to reduce the prevalence of stunting by 40% and wasting to <5% (5). However, the progress toward childhood malnutrition in developing countries has been deplorably slow. Globally, malnutrition among children under 5 years of age is estimated to contribute to more than one-third of all deaths, although it is rarely listed as the direct cause (6). The United Nations Children's Fund (UNICEF), WHO, and the World Bank Group reports recently revealed that globally, stunting affected an estimated 21.3% or 144 million children under the age of 5 years, and wasting continued to threaten the lives of an estimated 6.9%, or 47 million children under 5 years (7). Africa and Asia have the greatest burden of childhood undernutrition and account for 55 and 39% of global cases of undernutrition, respectively. In addition, more than half of all stunted children under 5 years live in Asia, whereas more than one-third live in Africa. More than two-thirds and more than one-quarter of wasted under 5 years of age live in Asia and Africa (8).

Adequate nutrition is essential for the healthy growth and development of children. The consumption of nutrients by children begins long before birth. Undernutrition during pregnancy stunts fetal growth and can lead to poor brain development, resulting in irreversible damage (9). During the growth period, especially in the first 5 years of a child's life, undernutrition can cause serious effects, such as wasting and stunting (10). In addition, undernutrition also has a negative effect on children's social skills and psychological development, such that underweight and stunted children are more likely to exhibit apathy, fewer positive emotions, and more insecure attachments (11). These children will have more problems with behavior, attention, and social relationships during their school years compared with non-stunted children (12). The intellectual and psychological deficits caused by undernutrition can persist into adolescence, which can negatively affect the nation's gross domestic product (13, 14). As a result, it is necessary to identify the underlying risk factors associated with malnutrition, based on the adopted WHO malnutrition framework model, so that governments and stakeholders can implement evidence-based policy and provide practical guidelines to improve childhood nutrition status (2).

Various studies have identified that low dietary intake, low birth weight, higher birth order, low parental education level, exclusive breastfeeding more than 6 months of age, illnesses such as diarrhea, and sex of child (male) are contributing factors to childhood undernutrition (15–18). However, the birth interval or the time interval between successive live births is a risk factor that has received little attention. According to a recent study conducted in 34 sub-Saharan countries, short birth intervals (<24 months) are strongly associated with childhood undernutrition and a 57% higher risk of infant mortality (19). Separate studies and reviews have also identified that short birth intervals could adversely affect the nutritional status of the mother and the child (20–23). However, the question of whether and to what extent long birth interval has on better nutritional outcomes in children remains unclear. Therefore, the present study aimed to carry out an in-depth analysis to evaluate different birth interval groups and child nutrition outcomes, including underweight, wasting, and stunting.

## Materials and methods

### Literature search strategies

This is a meta-analysis study and follows the Preferred Reporting Items for Systematic Reviews and Meta-Analysis (PRISMA) Guideline to examine the pooled odds ratio of the birth interval and its association with child undernutrition. A comprehensive literature search of research studies published before 30 June 2022 was conducted. Different search engines including PubMed, Web of Science, Science Direct, Google scholar, and Cochrane library were methodically searched. We tried to search for studies in different languages, but only English articles appeared to have relevant data about birth intervals and undernutrition, so we only used a single language for the search.

A further computerized search was conducted using a combination of medical subject headings or keyword terms for birth interval and child undernutrition and was used separately in combination using Boolean operators such as “OR” or “AND.” Terms for birth intervals included birth interval, birth spacing, interpregnancy interval, interbirth interval, preceding birth interval, and subsequent birth interval, and for the child undernutrition, these terms were also used: undernutrition, malnutrition, nutrition status, nutrition outcomes, child growth, stunting, wasting, and underweight.

### Inclusion and exclusion criteria

Studies included in this review had to meet the following criteria: “(1) cross-sectional, case–control, or cohort studies that evaluated the relationship between birth interval and any of the child undernutrition indicators, namely underweight, stunting, and wasting; and (2) original data were available. Studies were excluded if they were case series or reports, editorials, and reviews and if original data to calculate the association were unavailable.

### Data extraction

Two authors independently extracted all necessary data. The full text of these potentially eligible studies was retrieved and assessed for eligibility by two review team members. Any discrepancies were resolved jointly. The data extraction format included the first author's name, publication year, country, region, sample size, study design, interval group, undernutrition indicators measured, and the quality score of each study.

### Quality assessment

Two authors independently assessed the quality of the eligible studies and controlled for possible bias by adapting specific protocol/sample characteristics. The criteria proposed in the Newcastle–Ottawa Scale quality assessment tool were adapted and used to assess the quality of each study. Two authors independently assessed the quality of each original study using the tool. Discrepancies between the two authors were resolved jointly.

### Statistical analysis

The effect size of the meta-analysis was an odds ratio of underweight, stunting, and wasting reported in each study about the birth interval applied, the pooled odds ratio with a confidence interval of underweight, stunting, and wasting, according to the birth intervals grouped into (<24 and ≥24) were estimated. When heterogeneity between studies was absent, we merged the results using fixed effect models. Otherwise, a random-effects model was chosen. Subgroup analyses were conducted for the studies according to regions and by considering the birth intervals, which were classified into 24–48 against <24 and ≥48 against <24 to determine the extent to which a certain birth interval used is considered a risk factor or protective according to the nutrition outcomes (underweight, stunting, and wasting). To determine the extent of publication bias, funnel plots were scattered and tested for asymmetry, and Begg's tests were computed. The analysis was performed using STATA version 15 statistical software (24).

### Outcome definition

This study had three main outcomes; undernutrition was the main outcome and had three different indicators: underweight, stunting, and wasting. Each of the three indicators was measured independently according to WHO classification using child growth standard medians in terms of standard deviations (2). The first outcome was underweight, which was defined as a weight-for-age Z-score below minus two standard deviations (−2 SD) from the mean of the reference population. The second outcome was stunting, defined as a height-for-age Z-score of <-2 SD from the mean of the reference population. The third outcome was wasting, which was defined as a weight-for-height Z-score of <-2 SD from the mean of the reference population (25).

## Results

### Study selection

**Figure 1:**
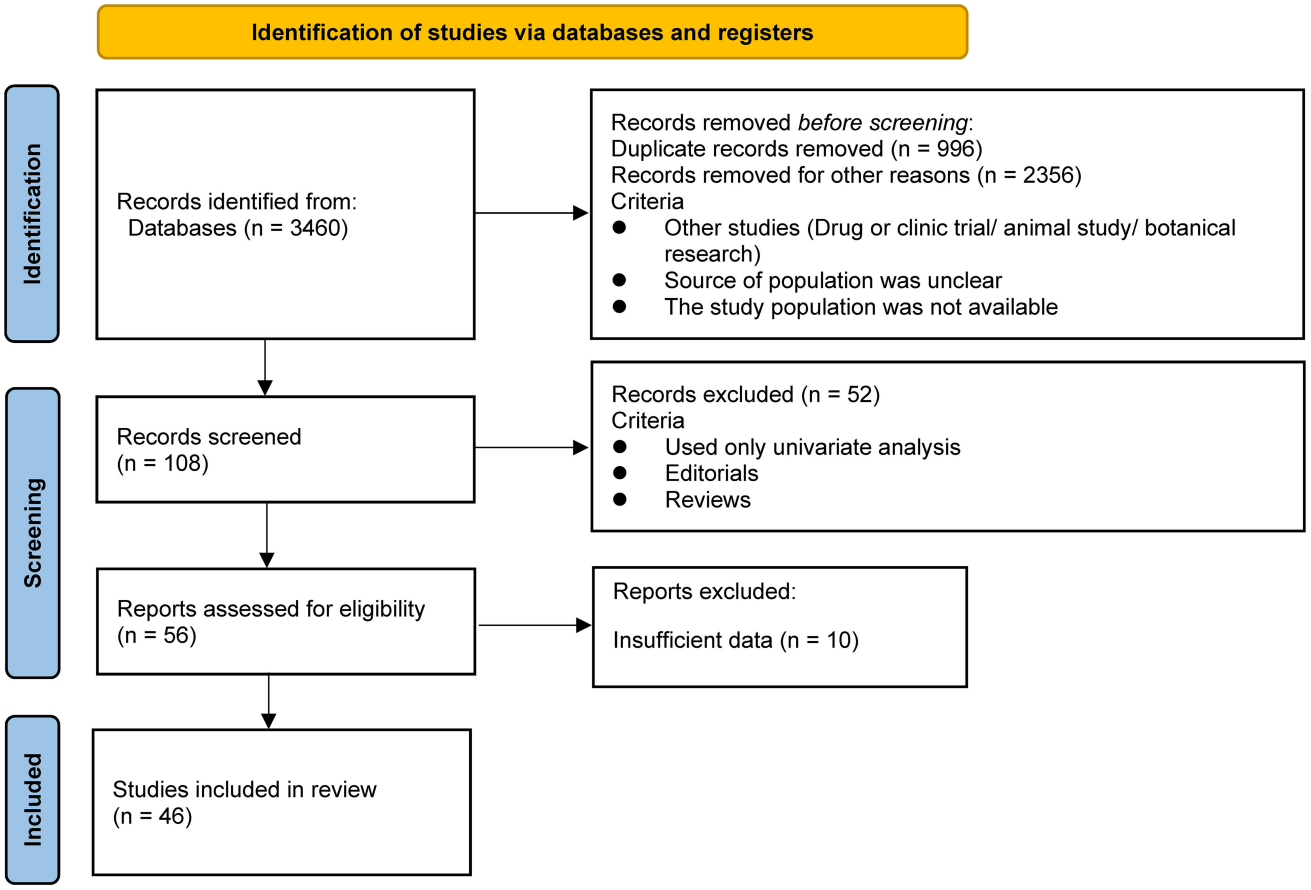
PRISMA flow diagram for the study selection process.

As shown in Figure 1, our literature search strategy identified 3,460 studies that were exported to the database; 3,352 studies were first excluded (996 of which were excluded because of duplication, 2,356 were excluded because the study type did not match, the population source was unclear, and the study population was not available), resulting in 108 studies with titles and abstracts screened for the relevance. Of these, 52 studies were removed because they used only univariate analysis or the type of literature was Editorials or Reviews. The remaining 56 relevant studies were evaluated, and 10 of them were excluded because of insufficient data on the relationship between birth spacing and child nutrition outcomes. Finally, 46 eligible studies were included in the analysis (1, 15, 19, 22, 23, 25–65). Regarding the child nutrition outcomes reported in the total 46 studies with data, 27 underweight data were reported, 23 stunting data were reported among all studies, and 13 wasting data were reported; seven studies reported all three undernutrition indicators in their result tables.

### Description of included studies

As shown in Table 1, 46 studies involving 898,860 children were included to examine the relationship between birth interval and child nutrition outcomes. Most of the included studies were cross-sectional studies; 32 of the 46 studies were demographic health survey (DHS)-based cross-sectional studies. Concerning the study regions, 18 studies were conducted in Africa, 20 studies in Asia, and eight studies in others. The quality score among the 46 included studies ranged from 6 to 9.

**Table 1:** Characteristics of literature included in the study.

**Number**	**First author**	**Year**	**Country**	**Region**	**Sample size**	**Study design**	**Interval group (months)**	**Undernutrition indicators reported**			**Quality**
								**Underweight**	**Stunting**	**Wasting**	**Scores**
1	Bater	2020	Uganda	Africa	3,337	DHS^b^	≤ 24; >24	√			8
2	Kahssay	2020	Ethiopia	Africa	269	Case-control	<24; ≥24		√		7
3	Yaya	2020	Multi^a^	Africa	171,371	DHS	<24; 24–47; ≥48		√		9
4	Das	2020	India	Asia	3,578	DHS	<24; ≥24		√		8
5	Ntenda	2019	Malawi	Africa	4,047	DHS	<24; 24–47; ≥48	√	√	√	8
6	Khatun	2019	Bangladesh	Asia	16,626	DHS	≤ 23; 24–47; ≥48		√	√	9
7	Takele	2019	Ethiopia	Africa	8,743	DHS	<24; 24–47; ≥48		√		8
8	Gupta	2019	Afghanistan	Asia	2,199	DHS	<24; 24–47; ≥48	√			7
9	Dessie	2019	Ethiopia	Africa	6,009	DHS	<24; 24–47; ≥48			√	8
10	Yaya	2019	Multi^a^	Africa	299,065	DHS	<24; 24–36; 37–59; ≥60	√	√	√	9
11	Fenta	2019	Ethiopia	Africa	7,830	DHS	<24; 24–35; 36–47; 48–59; ≥60		√		8
12	Fatemi	2018	Iran	Asia	172	Case-control	<24; 24–47; >48		√		7
13	Ansuya	2018	India	Asia	349	Case-control	<24; 24–47; >48	√			7
14	Talukder	2018	Bangladesh	Asia	7,102	DHS	<24; 24–47; >48		√		7
15	Talukder	2017	Bangladesh	Asia	7,102	DHS	<24; 24–47; ≥48	√			8
16	Remonja	2017	Madagascar	Africa	530	Case-control	<24; 24–47; ≥48		√		7
17	Pravana	2017	Nepal	Asia	277	Case-control	<24; ≥24			√	7
18	Kismul	2017	Congo	Africa	6,674	Case-control	<24; 24–47; ≥48		√		8
19	Mulugeta	2017	Ethiopia	Africa	321	DHS	<24; 24–48; >48	√	√	√	8
20	Darsene	2017	Ethiopia	Africa	811	DHS	<24; ≥24			√	7
21	Abera	2017	Ethiopia	Africa	342	DHS	<24; 24–35; 36–47; ≥48	√			7
22	Batiro	2017	Ethiopia	Africa	465	Case-control	≤ 24; >24		√		8
23	Olita'a	2014	Papua New Guinea	Oceania	68	Case-control	≤ 24; >24	√			7
24	Egata	2014	Ethiopia	Africa	2,199	Case-control	<24; ≥24			√	7
25	Khanal	2014	Nepal	Asia	3,490	DHS	<24; ≥24	√			8
26	Shahjada	2014	India	Asia	332	Case-control	<24; 24–48; >48	√	√	√	6
27	Adekanmbi	2013	Nigeria	Africa	2,8647	DHS	<24; ≥24		√		8
28	Ikeda	2013	Cambodia	Asia	7,453	DHS	<24; 24–47; ≥48	√	√	√	8
29	Sebayang	2012	Indonesia	Asia	8,568	Case-control	≤ 24; >24	√			9
30	Das	2011	Bangladesh	Asia	5,896	DHS	<24; 24–47; ≥ 48	√			8
31	Gribble	2009	El Salvador	America	3,852	DHS	<24; 24–35; 36–59; ≥60	√	√		8
32	Zottarelli	2007	Egypt	Africa	7,400	DHS	< 23; 24–35; 36–47; ≥48	√	√	√	8
33	Som	2007	India	Asia	2,835	DHS	<24; ≥24		√		8
34	Som	2006	India	Asia	1,186	DHS	<24; 24–47; ≥ 48	√	√	√	7
35	Hosain	2006	Bangladesh	Asia	227	DHS	<24; ≥24	√			7
36	Aerts	2004	Brazil	America	3,289	Case-control	<24; >24		√		8
37	Kurup	2004	Oman	Asia	1,198	Case-control	<24; >24	√			8
38	Mozumder	2000	Bangladesh	Asia	1,562	DHS	<24; 25–36; 37–48; ≥49	√			8
39	BP Zhu	1999	USA	America	173,205	DHS	<24; 24–59; 60–119; ≥120	√			9
40	Basso	1998	Denmark	Europe	10,187	DHS	<24; 24–36; >36	√			8
41	Basso	1998	Denmark	Europe	55,201	DHS	<24; 24–36; 36–60; >60	√			9
42	Adams	1997	Georgia	Europe	28,273	DHS	<24; 24–35; 36–47; ≥48	√			9
43	Hoa	1996	Vietnam	Asia	774	DHS	≤ 23; 24–35; ≥36	√			7
44	Huttly	1992	Brazil	America	3,586	DHS	<24; 24–35; 36–47; 48–71; >71	√			8
45	Thaver	1990	Pakistan	Asia	318	Case-control	<24; >24	√			7
46	Bertrand	1988	Zaire	Africa	1,895	DHS	<24; >24		√	√	8

a Including 34 sub-Saharan countries.

b Demographic health surveys.

### Quantitative synthesis

**Figure 2:**
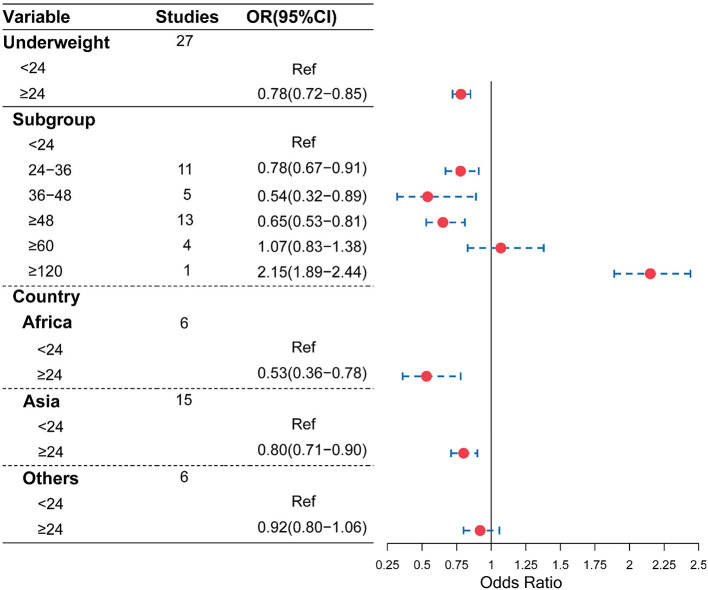
Association of the birth interval and child underweight.

**Figure 3:**
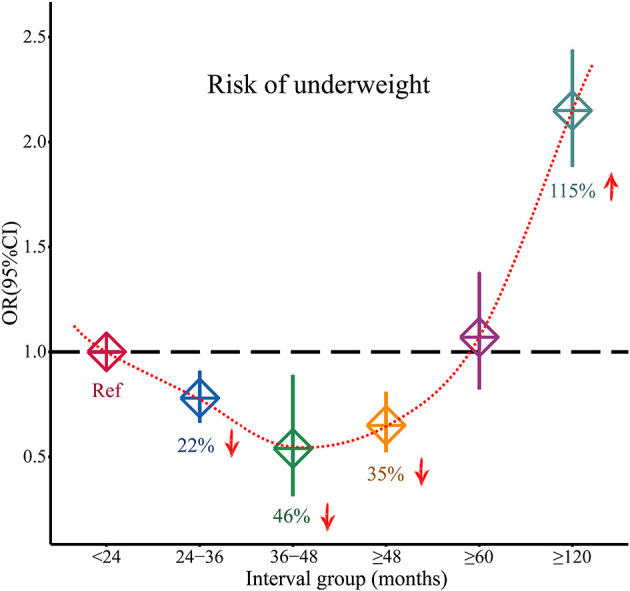
Changed effects of different birth interval groups and child underweight (<24 birth interval group was considered as the reference group; squares represent the ORs, and vertical lines represent the corresponding 95% CI).

As shown in Figure 2, compared with the birth interval of <24 months, the birth interval of ≥24 months was significantly associated with a decreased risk of being underweight (OR = 0.78, 95% CI = 0.72–0.85). Furthermore, when we further divided the birth intervals into subgroups, birth intervals and risk of being underweight showed a U-shape. As shown in Figures 2, 3, compared with the birth interval of <24 months group, the birth interval group of 24–36 months was significantly associated with a 22% decreased risk of being underweight (OR = 0.78, 95% CI = 0.67–0.91), while the group of 36–48 months was 46% more protective (OR = 0.54, 95% CI = 0.32–0.89). However, there was no protective effect in the group with a birth interval of ≥60 months when compared with the birth interval of <24 months (OR = 1.07, 95% CI = 0.83–1.38). Interestingly, in contrast, the birth interval group of ≥120 months was significantly associated with a 115% increased risk of being underweight when compared with the birth interval of <24 months (OR = 2.15, 95% CI = 1.89–2.44). Meanwhile, in the subgroup analysis based on regions (Figure 2), the protective effect was significant in Africans (OR = 0.53, 95% CI = 0.36–0.78) and Asians (OR = 0.80, 95% CI = 0.71–0.90).

**Figure 4:**
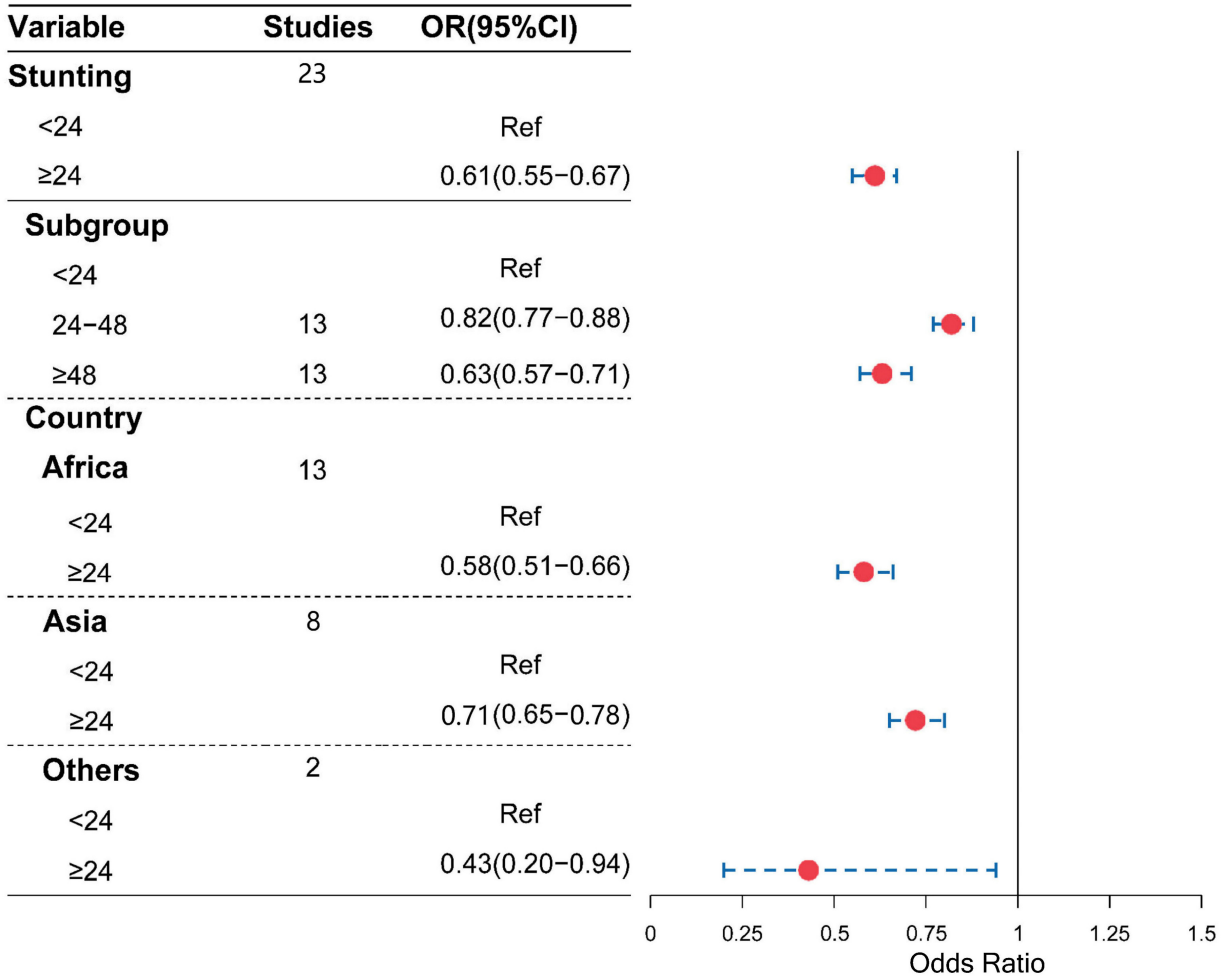
Association of birth interval and child stunting.

**Figure 5:**
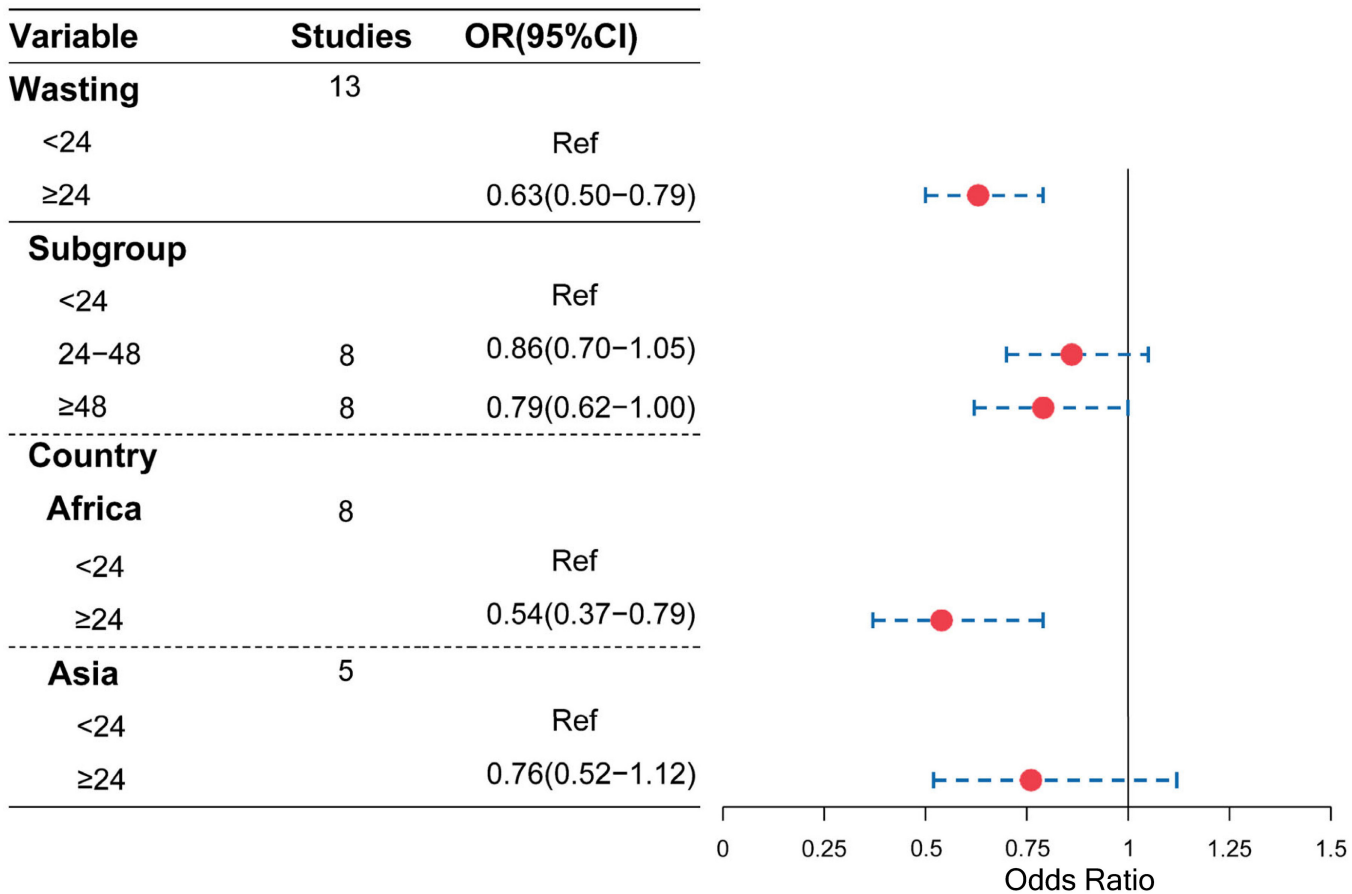
Association of birth interval and child wasting.

Moreover, as shown in Figures 4, 5, compared with a birth interval of <24 months, a birth interval of ≥24 months was significantly associated with a decreased risk of stunting (OR = 0.61, 95% CI = 0.55–0.67) and wasting (OR = 0.63, 95% CI = 0.50–0.79), respectively. We further divided the birth interval into subgroups, the birth interval group of <24 months was considered as the reference group, as shown in Figure 4, and the results showed that the birth interval group of 24–48 months was significantly associated with a decreased risk of stunting (OR = 0.82, 95% CI = 0.77−0.88), while the above 48 months groups yielded a clearer protective effect of stunting compared with a birth interval of <24 months (OR = 0.63, 95% CI = 0.57–0.71). In the subgroup analysis based on regions, the protective effects with a birth interval of ≥24 months for stunting were both significant in Africans and Asians, while similar results were observed for wasting in Africans.

### Publication bias

**Figure 6:**
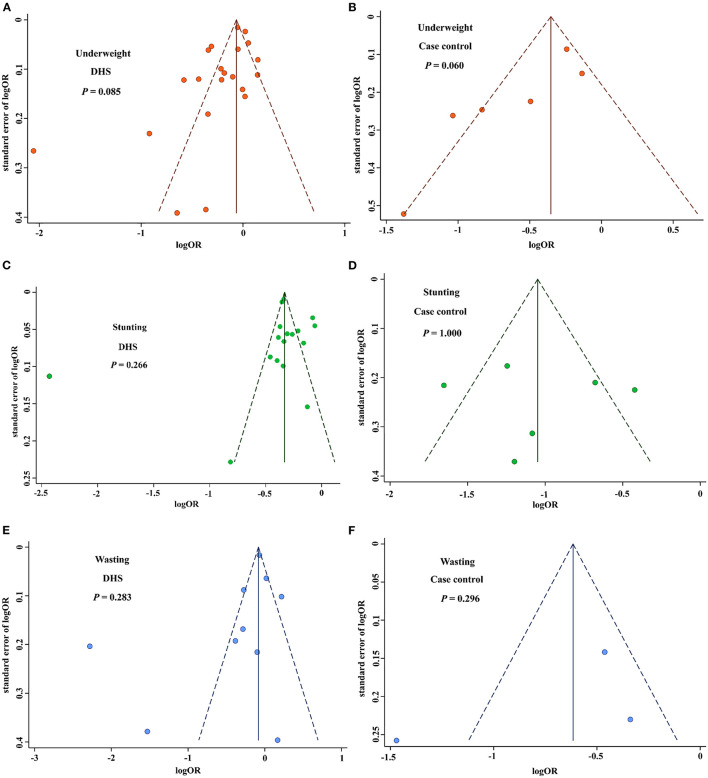
Funnel plot for publication bias of birth interval and childhood nutrition outcomes. **(A)** Funnel plot for publication bias of underweight based on the DHS studies. **(B)** Funnel plot for publication bias of underweight based on the case-control studies. **(C)** Funnel plot for publication bias of stunting based on the DHS studies. **(D)** Funnel plot for publication bias of stunting based on the case-control studies. **(E)** Funnel plot for publication bias of wasting based on the DHS studies. **(F)** Funnel plot for publication bias of wasting based on the case-control studies. DHS, demographic health surveys.

We then utilized the funnel plot and Begg's test to evaluate potential publication bias in the literature. The funnel plots were symmetrical in all the studied undernutrition outcomes (Figure 6). Moreover, Begg's test provided further statistical evidence for the absence of publication bias in all the studied undernutrition outcomes (*P* > 0.05).

## Discussion

The results of our study show that a longer birth interval (≥24 months) is significantly associated with a reduced risk of childhood undernutrition. Moreover, birth interval of ≥24 months and risk of underweight showed a U-shape that the optimum birth interval group of 36–48 months had the most protective effect compared with the birth interval of <24 months.

The most important risk factors for child undernutrition have been proven to occur early in life, including inadequate breastfeeding and maternal undernutrition during pregnancy (13). If longer birth intervals are maintained appropriately, more time will be provided for the care of older children, including the possibility of extended breastfeeding. The mother will also have time to recover from the nutritional burden of the last pregnancy, reducing the risk of undernutrition during the next pregnancy. Our study shows that an optimum birth interval between 36 and 48 months is independently associated with a significantly decreased risk of a child being underweight. Although the results cannot be directly compared with some studies due to different birth spacing boundaries and definitions, most studies report similar associations between birth spacing and poor child health outcomes. A cross-sectional study found that compared with the birth interval of >24 months, the risk of undernutrition was 1.43 times higher in children with birth intervals of <24 months (66). Moreover, a meta-analysis also reported a 3-fold increase in the odds of low birth weight for infants born <24 months apart (67). Generally, the short birth interval plays a major role in pregnancy outcomes, particularly among mothers with poor nutritional status, those with social-economic problems, and those with limited access to quality healthcare (68). The maternal nutrition depletion hypothesis states that a close sequence of pregnancies and periods of lactation worsens the mother's nutritional status (69, 70). This is because there is not enough time for the mother to recover from the physiological stresses of pregnancy before she is re-subjected to the stress. Our findings demonstrate that moderate birth intervals between 36 and 48 months would provide a mother with sufficient time to recover from the nutritional burden of pregnancy inherent during the prenatal period.

Some researchers have stipulated that having short birth intervals are caused by socioeconomic status, poorer lifestyles, failure to or inadequate use of healthcare services such as healthcare advice provided by healthcare advisers at community health centers, and other behavioral or physiological determinants, which will, in turn, lead to poorer pregnancy outcomes leading poor child nutrition outcomes (71, 72).

However, the effect of short birth intervals on children's nutritional status was not attenuated when socioeconomic and maternal characteristics were controlled (23, 73). This fact confirms that these confounding factors do not cause poor nutritional status endings, and short birth intervals are more likely to be an independent cause of poor nutritional status. Therefore, health policymakers should design appropriate policies to maintain desirable birth intervals, strengthen existing maternal and child nutrition interventions, and promote other relevant strategies to reduce child undernutrition, especially in developing countries.

This study had several strengths. First, our study presented a comparison between birth interval and all three undernutrition indicators: underweight, stunting, and wasting. Second, over 85% of the studies included in our meta-analysis were conducted in low-income and middle-income countries, which are mainly found in Africa and Asia, where the burden of child undernutrition is high. Therefore, this study supports the call to address the underlying causes of acute and chronic childhood undernutrition. Third, this study has identified areas where fellow researchers can design appropriate and strategic interventions to help the community to have healthy birth spacing based on our recommended birth interval.

There were some limitations to this study. First, there were few studies in developed countries, even though undernutrition is more prevalent in developing countries, so the results cannot be easily generalized. In addition, in many studies, assessing the relationship between birth interval and child nutritional outcomes was not a primary objective because birth interval was only one of the many variables examined. Most studies often lacked an assessment of all three nutritional indicators, namely, stunting, wasting, and underweight in their analysis. For three categories of child nutritional outcomes, further studies including a more comprehensive assessment of potentially confounding variables are needed to extract the complex factors involved in the relationship between birth interval and child nutritional outcomes.

## Conclusion

Our study reveals that a longer birth interval (≥24 months) is significantly associated with decreased risk of childhood undernutrition including underweight, stunting, and wasting. More importantly, the optimum birth interval of 36–48 months yielded the most protective effect for underweight, and this would allow for repletion prior to the next conception and conserves required nutrients for the baby's growth during and after the delivery, hence boosting further child nutrition status while reducing unacceptably high burden of child undernutrition.

## Data availability statement

The original contributions presented in the study are included in the article/supplementary material, further inquiries can be directed to the corresponding author.

## Author contributions

JN, WZ, AQ, and ZC contributed to the literature search and painting of all figures. JN wrote the manuscript. WZ contributed to the critical revision of the manuscript. MC designed the study and had full access to all data in the study and take full responsibility for the accuracy of the analyses and their interpretation. All authors read and approved the final manuscript.
